# Monitoring Match-Related Fatigue in Youth Rugby Players Using a Readiness Index and Clinical Tests

**DOI:** 10.3390/sports14070288

**Published:** 2026-07-08

**Authors:** Pierosario Giuliano, Daniela Vitucci, Daniele Pacini, Stefania Orrù, Annamaria Mancini

**Affiliations:** 1 Department of Medical, Exercise and Wellbeing Sciences, University Parthenope, 80133 Naples, Italy; stefania.orru@uniparthenope.it (S.O.); annamaria.mancini@uniparthenope.it (A.M.); 2 Department of Economics, Law, Cybersecurity and Sport Sciences, University Parthenope, 80035 Naples, Italy; daniela.vitucci@uniparthenope.it; 3 CEINGE-Biotecnologie Avanzate Franco Salvatore, 80145 Naples, Italy; 4 Performance Department, Federazione Italiana Rugby, 00135 Rome, Italy; d.pacini@federugby.it

**Keywords:** fatigue monitoring, load management, rugby, youth athletes, performance, readiness index

## Abstract

Monitoring athlete readiness in youth rugby players is important for understanding short-term responses to match demands and supporting recovery management. Here, we aimed to investigate short-term readiness by integrating subjective measures, clinically relevant tests, and match load data. This single-team study considered 28 male rugby players (17.8 ± 0.2 years), monitored every four consecutive days (match day [MD], MD+1, MD+2, MD+3). Readiness was assessed using four subjective dimensions (fatigue upon waking, mood, sleep quality, and muscle soreness), which were normalized to individual best values and combined into a composite readiness index (4-dRI; 0–1). The Adductor Squeeze Test (AST), Sit-and-Reach Test (SRT), and Global Positioning System-derived metrics (MD only) were also assessed. The 4-dRI decreased by approximately 30% at 24 h post-match (*p* < 0.001), indicating a substantial reduction in perceived readiness. Athletes exposed to higher GPS-derived match loads reported lower readiness on MD+1. AST showed moderate associations with the 4-dRI (ρ = 0.40, *p* < 0.05), whereas SRT appeared less responsive to short-term changes in readiness. These findings indicate that both the 4-dRI and AST were responsive to short-term changes in readiness across the competition period. Overall, these integrated data may contribute to athlete-monitoring strategies in team sport settings.

## 1. Introduction

The match demands of professional rugby teams have led rugby unions and federations to develop new projects and organize competitions aimed at improving the quality of high-level games and training. As a consequence, rugby clubs and associations have increased the hours of both training sessions and physical preparation to enhance match performance. However, performance enhancement often comes with a higher risk of injury [1,2].

The high physical demands of the game, together with frequent collisions and repeated eccentric muscle contractions associated with acceleration and deceleration, lead to increased markers of muscle damage and acute neuromuscular and perceptual fatigue after matches [3]. These effects may persist for several days following competition [3,4], contributing to a substantial risk of injury [2,5,6]. Recent systematic reviews indicate that injury rates in Rugby Union are approximately 20 injuries per 1000 player hours for time-loss injuries and can exceed 40 injuries per 1000 player hours during match play, with higher values observed in professional players [7].

During rugby competitions, particularly within development programs for young international athletes, it is essential to plan training sessions and organize strength and speed programs while taking injury prevention into account [1,8].

Consequently, the accurate quantification of match and training demands has become increasingly important for professionals working in rugby environments [9]. The Global Positioning System (GPS) tracking device has become a key tool for quantifying the external load experienced by athletes during training and matches [10,11,12]. GPS-derived metrics such as total distance, high-speed running efforts, and movement demands provide valuable information regarding the physical requirements of competition [10,11,12].

Previous research by Gabbett and colleagues demonstrated that injury risk is positively associated with increases in externally measured workload, particularly when GPS-derived running demands and total distance covered show large spikes over short periods of time [5,9]. These findings highlight the importance of monitoring load-related variables to better understand athletes’ responses to training and competition.

However, such measures alone may not adequately reflect the athlete’s overall condition [10,11,13,14,15,16,17,18]. The cumulative fatigue associated with frequent competitions and chronic training load is a well-recognized aspect of modern coaching practice [19,20,21], highlighting the need for effective load management strategies. According to Taylor et al., various methods are used to monitor fatigue in high-performance environments, although best practice remains unclear [22], and many coaches rely on subjective measures, particularly for injury prevention purposes, often using self-designed questionnaires focusing on muscle soreness, physical fatigue, and general wellness [4,20,22,23,24,25]. Nevertheless, fatigue is a multidimensional construct involving not only physiological but also psychological and behavioral factors [26,27]. Subjective perception plays a key role in reflecting the athlete’s internal state [24], which can be influenced by mechanisms such as sleep, recovery, and stress responses [28,29]. For instance, sleep deprivation may alter psychological responses related to fatigue [23,28,29].

Although subjective measures are considered sensitive indicators of fatigue and load-related changes, they may not fully capture the complexity of the athlete’s condition [20,24,30,31]. Therefore, objective assessments may complement subjective monitoring and provide additional information regarding physical readiness [20,31,32]. For example, the Adductor Squeeze Test is widely used in rugby as a clinical tool to assess adductor strength and monitor injury risk, with position-specific normative values reported in professional players [33,34]. However, evidence in youth populations remains limited. Similarly, the Sit-and-Reach Test is commonly used to assess hamstring flexibility and lower back mobility [35,36,37], showing high reliability and acceptable validity [37,38].

In this context, the use of simple clinical and field-based tests represents a practical and non-invasive approach to assess physical condition and indirectly monitor readiness [33,34,35,36,37]. However, the application of these tests for monitoring fatigue and readiness in youth rugby players is scant. Previous studies have examined either subjective wellness measures or GPS-derived external load metrics in rugby players [9,11,17,18]. However, evidence integrating subjective readiness dimensions, simple clinical field tests, and match-load characteristics within the same monitoring framework remains limited, particularly in youth international rugby settings [20,24,30]. Furthermore, it is unclear whether a composite readiness score can effectively detect short-term post-match fatigue responses [39]. Such an approach may provide a more practical representation of overall readiness rather than limited knowledge derived from individual dimensions of wellness.

The aim of the present study was to assess the readiness of young rugby players during the Six Nations Under-18 Festival (FSN18) by exploring four specific dimensions (fatigue upon waking, FW; mood, Mo; sleep quality, SQ; and muscle soreness, MS) in relation to GPS tracking data collected on match day (MD) and two clinical tests (CTs). A further objective was to test a novel four-dimensional readiness index (4-dRI), combining these dimensions into a single normalized score, as a practical tool for monitoring short-term fatigue responses in youth rugby players. It was hypothesized that athletes would reach peak readiness on MD, with the lowest levels occurring 24 to 48 h later, indicating a fatigued state.

## 2. Materials and Methods

### 2.1. Participants and Six Nations Under 18 Festival

This study, based on a single-team design, was conducted on 28 international U18 athletes (age: 17.8 ± 0.2 years; body weight: 88.30 ± 11.92 kg; height: 184.54 ± 7.45 cm) during a high-level international youth rugby competition (FSN18), a highly demanding ten-day competition during which each team played three matches with an average recovery period of approximately three days between games. All teams competed on the same days, within a consistent time window (12:00–15:00). A mandatory rotation rule ensured that each athlete started in at least one of the three matches. All participants played an average of 147.5 ± 71.6 min throughout the tournament. All 28 players included in the match-day squad participated in the monitoring procedures and completed the entire tournament. No athlete withdrew from the study or missed the monitoring period due to injury, illness, or other reasons. Prior to the festival, a five- to six-day training camp was organized, involving 32 selected players, of whom 28 were included in the official match-day squad. Therefore, inclusion criteria were selection in the FSN18 match-day squad and availability for the monitoring procedures throughout the tournament. The data were collected as part of routine athlete monitoring in collaboration with the Italian Rugby Federation (FIR).

The athletes were selected by the Italian Rugby Federation from the U18 players regularly competing in the national U18 championship. All players attended the federation’s high-performance development centers and participated in training camps and international preparation activities before the FSN18 competition. These activities ensured that the athletes were familiar with the testing and monitoring procedures included in the experimental protocol. Preparation and performance data from the first match were deliberately excluded due to their different nature compared with subsequent matches.

### 2.2. Study Design

The readiness of young rugby players was assessed during the FSN18 by exploring four specific dimensions (fatigue upon waking, FW; mood, Mo; sleep quality, SQ; and muscle soreness, MS) in relation to GPS tracking data collected on match day (MD) and two clinical tests (CTs).

To assess the readiness of young rugby players, both subjective and objective measures were used. The subjective measures provided key indicators regarding FW, Mo, SQ, and MS. The objective measures consisted of flexibility and strength evaluations by means of two clinical tests [33,36]. Additionally, external load was assessed using GPS tracking devices (K-Sport, Pesaro, Italy; 50 Hz GNSS and 283 Hz accelerometer) [17,40]. GPS tracking data were exclusively collected on match day (MD), whereas subjective and objective measures were collected daily during the ten-day competition.

**Figure 1:**
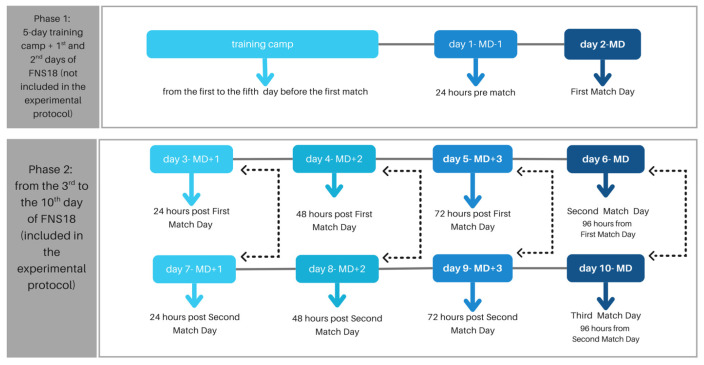
Schematic representation of the days the athletes spent in the training camp and during the FSN18. Training camp days allowed athletes to familiarize themselves with the experimental procedure. Subjective and objective measurements collected during Phase 2, after the first match (day 2, MD+1), were used in this study to evaluate athletes’ readiness.

These assessments were performed on four consecutive days for two matches during the festival, according to the scheme shown in Figure 1: 24 h post-match (MD+1), 48 h post-match (MD+2), 72 h pre-match (MD+3), and on match day (MD), 96 h after the previous match. These specific time points were selected to capture the acute responses to match-related load and the recovery process in the days following and preceding the competitions (Figure 1).

### 2.3. The Subjective Components

The subjective components of readiness were assessed by means of a daily questionnaire, delivered via Google Forms, probing FW, Mo, SQ, and MS dimensions [29]. Each of them provides insights into the athlete’s physical and mental state and was evaluated using a 5-point Likert scale (rated from 1 = very poor to 5 = very good) [41].

The questionnaire was administered early in the morning, before breakfast, in a dedicated room where all athletes completed the daily assessment. Data collection started before the festival during pre-festival training camps (Figure 1), during which athletes were familiarized with the entire protocol, including both questionnaires and clinical tests. During these sessions, the highest value recorded for each parameter was identified as the individual best score. These values were considered stable and representative peak references and were subsequently used as fixed values for normalization. No further updates of these reference values were performed during the competition period. A simplified version of the questionnaire proposed by Carlos Ramirez Lopez et al. was used [29]; the questionnaire is available as Supplementary Materials (Figure S1).

### 2.4. The Objective Components

The objective components included two clinical tests (CTs): the Adductor Squeeze Test (AST), used to evaluate the risk of developing groin pain by monitoring adductor muscle strength, a known risk factor for groin injuries in team-sport athletes and commonly applied in rugby-specific screening [33,34,42], and the Sit-and-Reach Test (SRT), used to measure hamstring muscle extensibility, with flexibility being a key parameter for preventing muscle injuries and knee pain [36,37,38].

Briefly, in the AST, the athlete lies supine, with hips and knees at 90° without support; the sphygmomanometer cushion is placed between the knees and 100 mmHg of pressure is applied. The sphygmomanometer was positioned and monitored by the examiner, while pressure values were recorded directly from the device. The athlete then performs an adduction movement by squeezing the thighs together, holding for 5 s. The maximum pressure reached is recorded. If the athlete experiences pain, the pressure value at the onset of pain is noted by the operator.

In the SRT, the athlete sits with buttocks and shoulders against a wall, and hands stacked on a graduated scale; as the goal is to measure the furthest point the hands can reach, the athlete bends forward, keeping the legs straight for 3 s, and the operator records the distance reached (in cm). As reported above, the two tests were conducted every morning, before breakfast, in a dedicated room (Supplementary Materials, Figure S2). Athletes had been familiarized with both clinical testing procedures during the pre-festival training camp as part of the experimental protocol (Figure 1). All AST and SRT assessments were performed by the same experienced examiner throughout the study period in order to ensure consistency of measurement procedures.

### 2.5. GPS Tracking

During match days (MD), all players wore a GPS tracking device provided by K-Sport (K-50 Wearable Tech, with a sampling rate of 50 Hz). The physical demands of young players in rugby matches are reflected by the relative distance covered over time and running speed at different intensities [1,12,43]. These metrics were chosen due to their relevance in quantifying external load [5]. Hence, although the GPS recorded multiple metrics, the variables selected for this experimental protocol were minutes played (MP), total distance (TD), and the number of efforts performed at speeds over 20 km/h (nEF). Following each match, GPS data were downloaded from the devices and imported into the K-Sport proprietary analysis software (K-Sport, Italy). Using the software, individual match files were manually trimmed according to the exact start and end times of each match, excluding warm-up activities and half-time periods. Processed data were subsequently exported in CSV format and used for statistical analyses. All GPS recordings were successfully acquired and no missing GPS data were observed for the matches included in the study.

### 2.6. Indexing and Aggregate Readiness Index

To monitor the athlete’s readiness state, each subjective and objective component was used to calculate daily normalized indices (Y, ranging from 0 to 1), including fatigue upon waking (*Y_FW_*), mood (*Y_Mo_*), sleep quality (*Y_SQ_*), muscle soreness (*Y_MS_*), Adductor Squeeze Test (*Y_AST_*), and Sit-and-Reach Test (*Y_SRT_*). Each index was calculated by dividing the athlete’s daily score (xi) by the individual reference value (Xj), defined as the personal best obtained during the pre-festival training period [39].

Y = xi/Xj

This normalization process allowed the transformation of all variables into comparable dimensionless indices, enabling their aggregation.

The four-dimensional readiness index (4-dRI), representing the subjective component only, was computed as the arithmetic mean of the normalized indices (*Y_FW_*, *Y_Mo_*, *Y_SQ_*, *Y_MS_*) [44]. All variables were assigned equal weight in the computation of the composite index, as no established evidence or validated weighting scheme is currently available to support differential weighting of fatigue upon waking, mood, sleep quality, and muscle soreness in youth athletes. These indices provide a daily assessment of the athlete’s readiness and may support load management strategies during the competition period.

### 2.7. Statistical Analysis

Statistical analyses were performed using JASP (version 0.95.4) and R (version 4.3.1). Descriptive statistics are presented as mean ± standard deviation (SD) for continuous variables and as median with interquartile range (IQR) for ordinal or non-normally distributed variables. Prior to inferential analyses, data distribution was assessed using the Shapiro–Wilk test. The preliminary analysis indicated that several subjective and objective variables violated the assumption of normality and/or were ordinal in nature. To account for the hierarchical structure of the data arising from repeated measures nested within participants, and given the non-normal distribution of the raw scores, linear mixed-effects models (LMMs) were implemented. LMMs provide a robust analytical framework that accommodates clustered data structures and mitigates the limitations of traditional parametric tests regarding normality violations.

Although the original questionnaire responses were collected using ordinal Likert scales, all subjective variables were transformed into normalized indices ranging from 0 to 1 and were therefore treated as continuous outcomes in LMMs.

To investigate differences in subjective and objective readiness indices across time points (Match Day [MD], MD+1, MD+2, and MD+3), LMMs were applied.

Separate models were fitted for each dependent variable: FW index (*Y_FW_*), Mo index (*Y_Mo_*), SQ index (*Y_SQ_*), MS index (*Y_MS_*), AST index (*Y_AST_*), and SRT index (*Y_SRT_*). MD was included as a categorical fixed factor. Degrees of freedom and statistical significance of fixed effects were assessed using the Satterthwaite Type III approximation in JASP (version 0.95.4). When a significant main effect of MD was observed, post hoc pairwise comparisons were conducted on estimated marginal means using treatment contrasts, with MD set as the reference level, to enable direct comparisons between MD and subsequent days (MD+1, MD+2, and MD+3). Pairwise comparisons were adjusted for multiple testing using the Bonferroni correction. Statistical significance was set at *p* < 0.05, *p* < 0.01, and *p* < 0.001.

The same rationale regarding ordinal and non-normally distributed data was applied to correlation analyses. Spearman rank correlations (rho) were computed to examine relationships between subjective and objective variables, as well as between match load and readiness indices. In addition, correlations between individual subjective components and the composite 4-dRI were examined to explore their relative contribution to the overall index. The absolute values of correlation coefficients were interpreted as trivial (|rho| < 0.30), small (0.30 < |rho| < 0.50), moderate (0.50 < |rho| < 0.70), and strong (|rho| > 0.70). Correlation analyses between readiness variables and time points (MD, MD+1, MD+2, MD+3) were computed separately, while those between GPS metrics and subjective variables were restricted to MD and MD+1 to capture the acute post-match response. Spearman’s correlation heatmaps were generated using JASP. To supplement the correlation framework, additional exploratory analyses were performed using Locally Weighted Scatterplot Smoothing (LOWESS) curves. This graphical approach was used to visually explore the potential non-linear relationship between GPS-derived load metrics and the normalized indices (*Y_FW_*, *Y_Mo_*, *Y_SQ_*, *Y_MS_*, *Y_AST_*, *Y_SRT_*) at the aggregated MD and MD+1 time points. All graphical representations related to the LMMs and LOWESS exploratory analyses were generated in R using the ggplot2 and patchwork packages.

## 3. Results

### 3.1. Analysis of the Subjective Components

**Figure 2:**
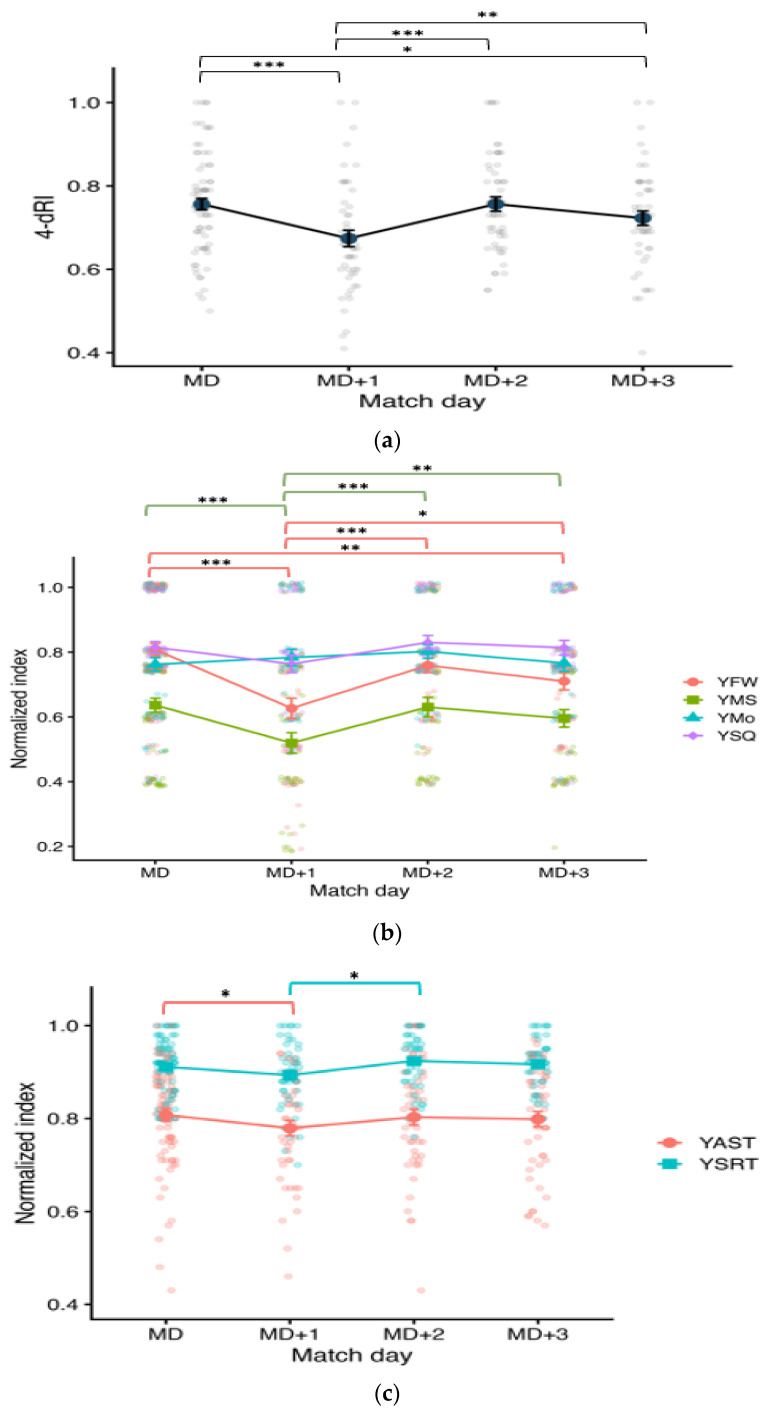
Distribution of readiness indices across match days. (**a**) Four-dimensional readiness index (4-dRI); (**b**) subjective indices; (**c**) objective indices. Dots represent individual observations, whereas colored points and error bars represent mean ± standard error. Abbreviations: MD, match day; *Y_FW_*, normalized fatigue upon waking; *Y_Mo_*, normalized mood; *Y_SQ_*, normalized sleep quality; *Y_MS_*, normalized muscle soreness; *Y_AST_*, normalized Adductor Squeeze Test; *Y_SRT_*, normalized Sit-and-Reach Test. Asterisks indicate significant pairwise comparisons derived from the linear mixed-effects models, (* *p* < 0.05, ** *p* < 0.01, *** *p* < 0.001).

The estimated marginal means derived from the linear mixed-effects model for all subjective variables during the days analyzed in the FSN18 are reported in Figure 2a,b; it is noteworthy that 4-dRI, *Y_FW_*, *Y_SQ_*, and *Y_MS_* are lower on MD+1, except for *Y_Mo_*, which is quite stable during the overall time course. All descriptive statistics of the subjective and objective components are displayed in Supplementary Materials Table S1.

The linear mixed-effects model revealed a significant main effect of match day on 4-dRI (F(3, 42.18) = 10.64, *p* < 0.001). Post hoc pairwise comparisons indicated that 4-dRI was significantly lower on MD+1 compared with MD (estimate = −0.09, *p* < 0.001). MD+1 was also significantly lower than MD+2 (estimate = −0.09, *p* < 0.001) and MD+3 (estimate = −0.06, *p* = 0.003). No significant differences were observed between MD and MD+2 (*p* = 1.000) or between MD+2 and MD+3 (*p* = 0.051). All pairwise comparisons are reported in Table 1. Additionally, linear mixed-effects models revealed a significant main effect of match day for *Y_FW_* and *Y_MS_* (F(3, 42.62) = 10.72, *p* < 0.001 and F(3, 49.03) = 8.314, *p* < 0.001, respectively), whereas no significant main effect was observed for *Y_Mo_* or *Y_SQ_* (both *p* > 0.05). Post hoc pairwise comparisons showed that *Y_FW_*, was significantly lower on MD+1 compared with MD (*p* < 0.001), and remained lower compared with MD+2 (*p* < 0.001) and MD+3 (*p* = 0.033). Similarly, *Y_MS_* values were significantly reduced on MD+1 compared with MD (*p* < 0.001), and were also lower than MD+2 (*p* < 0.001) and MD+3 (*p* = 0.008). No significant pairwise differences were detected across match days for *Y_Mo_* and *Y_SQ_* (all *p* > 0.05). All post hoc comparisons are reported in Table 1.

Spearman’s rank correlation (ρ) analysis showed strong, significant, and comparable associations between each individual dimension and the overall 4-dRI (ρ = 0.498–0.739, *p* < 0.001; see Table 2 for details), suggesting that each component contributes similarly to the composite index.

**Figure 3:**
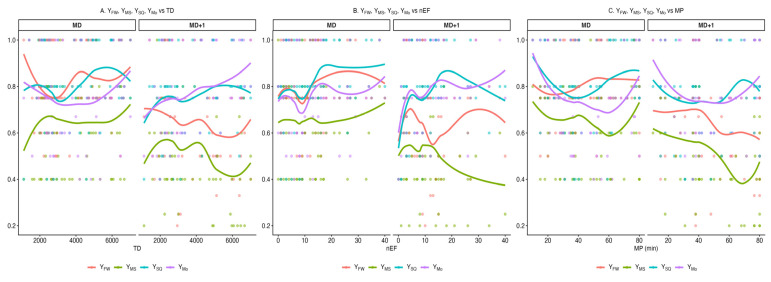

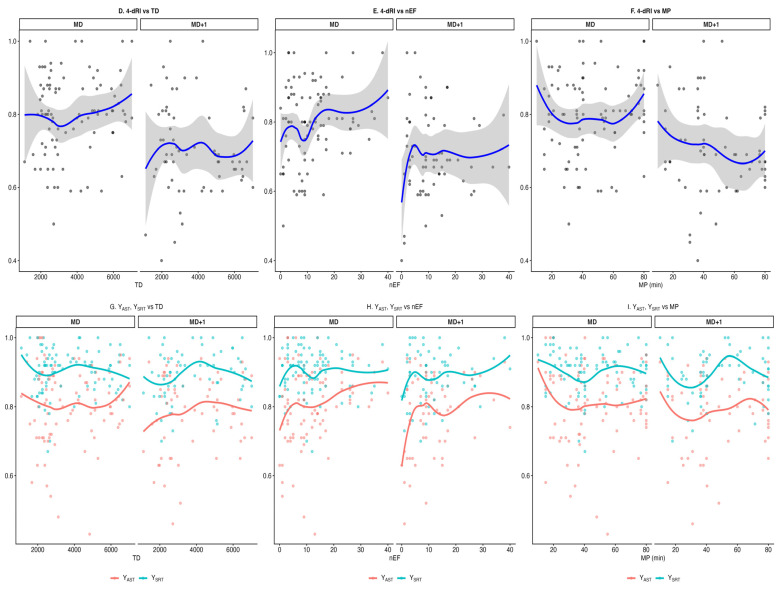
Locally weighted scatterplot smoothing (LOWESS) graphs illustrating the relationships between GPS-derived data collected on MD and subjective readiness indices and clinical test measures on MD and MD+1: *Y_FW_*, *Y_MS_*, *Y_SQ_*, and *Y_Mo_* vs. TD (**A**), nEF (**B**), and MP (**C**); 4-dRI vs. TD (**D**), nEF (**E**), and MP (**F**); YAST and YSRT vs. TD (**G**), nEF (**H**), and MP (**I**). Shaded areas represent 95% confidence intervals.

The flexplots in Figure 3A–F illustrate the relationship between match GPS-derived metrics and the normalized indices (*Y_FW_*, *Y_Mo_*, *Y_SQ_*, and *Y_MS_*) as well as the composite readiness index (4-dRI). On MD+1, the curves suggest non-linear associations between external load and perceived readiness. Notably, inflection points observed in the LOWESS curves may represent potential thresholds in the relationship between external load and perceived readiness, where further increases in load are associated with more pronounced reductions in perceived readiness.

### 3.2. Analysis of the Objective Components

Linear mixed-effects models showed a significant main effect of match day for both *Y_AST_* (F(3, 38.93) = 4.449, *p* = 0.008) and *Y_SRT_* (F(3, 32.80) = 3.101, *p* = 0.040). Post hoc analyses revealed that *Y_AST_* was significantly higher on MD compared with MD+1 (*p* = 0.013), whereas no other pairwise differences were observed across match days (all *p* > 0.05). For *Y_SRT_*, a significant difference was found between MD+1 and MD+2, with lower values on MD+1 (*p* = 0.041), while all remaining comparisons were not significant (all *p* > 0.05). Pairwise contrasts are reported in Table 3.

**Figure 4:**
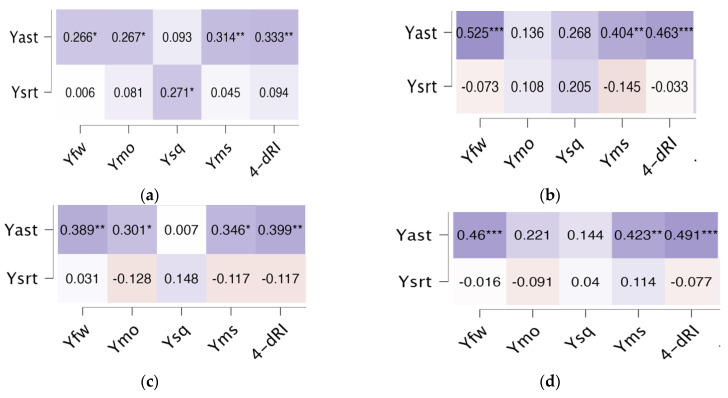

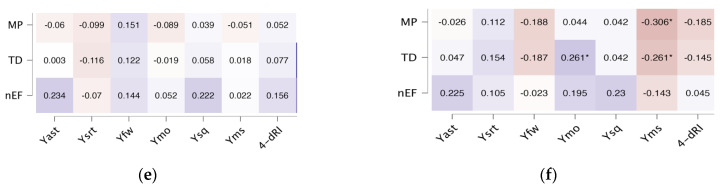
Spearman’s rho heatmaps showing correlations between subjective and objective indices on MD (**a**), MD+1 (**b**), MD+2 (**c**), and MD+3 (**d**), and between subjective indices and GPS-derived external oad variables on MD (**e**) and MD+1 (**f**). Abbreviations: TD, total distance; nEF, number of high-speed efforts (>20 km·h^−1^); MP, minutes played. * *p* < 0.05, ** *p* < 0.01, *** *p* < 0.001. Color intensity indicates the direction and magnitude of Spearman’s rho. Darker shades indicate stronger correlations, whereas lighter shades indicate weaker correlations.

Spearman’s rho correlations (ρ) between subjective and objective readiness indices are shown in Figure 4a–d. On MD, moderate to strong positive correlations were observed between *Y_AST_* and *Y_FW_*, *Y_Mo_*, *Y_MS_*, and the 4-dRI (ρ = 0.26, 0.31, and 0.33; *p* < 0.05, *p* < 0.05, and *p* < 0.01, respectively). On MD+1, *Y_AST_* remained moderately correlated with *Y_FW_*, *Y_MS_* and 4-dRI (ρ = 0.52, 0.40, and 0.46; *p* < 0.001, *p* < 0.01, and *p* < 0.01, respectively). Furthermore, other small associations were observed between *Y_AST_* and *Y_FW_*, *Y_Mo_*, *Y_MS_*, and 4-dRI on MD+2 (ρ = 0.38, 0.30, 0.34, and 0.39; *p* < 0.001, *p* < 0.05, *p* < 0.05, and *p* < 0.01, respectively), whereas on MD+3, significant moderate correlations were again detected between *Y_AST_* and *Y_FW_*, *Y_MS_*, as well as the 4-dRI (ρ = 0.46, 0.42, and 0.49; *p* < 0.001, *p* < 0.01, and *p* < 0.001, respectively). No consistent or significant correlations were found between *Y_SRT_* and subjective indices across match days, while no significant correlations were found between the CTs and the GPS metric data; all details are reported in Figure 4e,f. Additionally, flexplot analysis of *Y_SRT_* revealed patterns consistent with the differences between MD and MD+1, also identified by the linear mixed-effects models, particularly for TD metrics (Figure 3G). In contrast, the LOWESS curves for *Y_SRT_* did not show a clear separation between the two time points.

### 3.3. Analysis of the GPS Tracking

Spearman’s rho correlations (ρ) were used to examine the relationships between subjective readiness indices (*Y_FW_*, *Y_Mo_*, *Y_SQ_*, *Y_MS_*, and 4-dRI) and GPS-derived variables (total distance [TD], number of speed efforts > 20 km/h [nEF], and minutes played [MP]).

On MD, correlations between GPS variables and normalized indices (*Y_FW_*, *Y_Mo_*, *Y_SQ_* and *Y_MS_*), as well as the composite readiness index (4-dRI), were generally small and non-significant. TD and nEF showed weak associations with overall subjective indices, indicating limited relationships between external load and perceived or measured readiness on the same day (Figure 4e). Conversely, on MD+1, several small but significant correlations emerged (Figure 4f). TD was significantly and negatively correlated with *Y_MS_* (ρ = −0.26, *p* < 0.05), whereas nEF showed a negative but non-significant correlation with *Y_MS_* (ρ = −0.143, *p* > 0.05). MP also showed small negative correlations with *Y_MS_* (ρ = −0.30, *p* < 0.05; Figure 4f).

Additionally, visual inspection of the scatterplots with LOWESS suggested non-linear relationships between selected GPS metrics and normalized indices (*Y_FW_*, *Y_Mo_*, *Y_SQ_* and *Y_MS_*), as well as the composite readiness index (4-dRI) (Figure 3A–F). In particular, the trend lines showed a tendency toward lower readiness values after specific thresholds of external load were reached. For *Y_MS_*, an initial increase or stability was observed at lower values of neuromuscular events (nEF), followed by a gradual decrease at higher nEF values (Figure 3B). A similar pattern was observed for MP, where *Y_MS_* values tended to decline as playing time increased (Figure 3C). Regarding 4-dRI, the trend line showed a decrease at moderate-to-high MP, followed by a slight stabilization at the highest values (Figure 3F). Comparable patterns were observed for TD, where *Y_MS_* values increased at low-to-moderate distances but tended to decrease once higher distances were reached (Figure 3A). Additionally, visual inspection of the clinical variables revealed that *Y_AST_* showed patterns consistent with the differences observed between MD and MD+1, particularly in relation to TD metrics (Figure 3G), whereas *Y_SRT_* did not exhibit a clear trend or separation between the two time points.

## 4. Discussion

### 4.1. Subjective Components

This study demonstrates that an athlete’s state of readiness can be linked to specific subjective dimensions (FW, MS, Mo, SQ) during competition periods. The 4-dRI, measured on MD and the following day (MD+1), shows significant differences and may represent a useful tool for monitoring internal load 24 h after the match. Furthermore, as the next match approaches (MD+2 and MD+3), the values of 4-dRI and overall normalized indices progressively increase, peaking again on MD. In particular, as shown in Figure 2a, the 4-dRI on MD+1 falls below 0.7, suggesting that athletes may experience a greater fatigue state 24 h after match performance. The 4-dRI on MD+1 was approximately 30% lower than the athletes’ best scores and significantly lower than values observed on MD, indicating a substantial reduction in perceived readiness 24 h after competition. The observed pattern of correlations among the components was compatible with the equal-weighting approach adopted for the 4-dRI [44,45]. Moderate to strong associations (*p* < 0.05) between dimensions suggest that each component captures related but distinct aspects of readiness, while their strong correlations with the 4-dRI were consistent with their contribution to the composite score. According to Carlos Ramirez Lopez et al., comparing daily values with previously recorded maximum values helps determine the athletes’ readiness state [25]. In fact, the 4-dRI was used to monitor both the load trend during FSN18 and the athletes’ daily readiness. In addition, these findings further indicate that MS and FW, on MD+1, may serve as the most responsive 4-dRI indicators, both being negatively correlated with GPS tracking data (MP, TD, and nEF). MS appeared to be one of the most responsive dimensions for detecting post-match fatigue-related changes. The estimated mean of *Y_MS_* on MD+1 is 50% lower compared with athletes’ best scores and is significantly different from the same index detected on MD (Figure 2b). Internal load is defined as the biological and psychological stress imposed by external loads, or rather the effect of peaks in external load [46]. This latter induces changes in biochemical parameters such as DOMS (Delayed Onset Muscle Soreness), suggesting that the most appropriate indicator of internal load may indeed be the subjective perception of muscle soreness [1,5,40]. Current data support the idea that *Y_MS_* has greater sensitivity in detecting responses to internal load [43,47]. Similarly, the FW dimension also appears to be adequate for monitoring athletes’ fatigue and readiness, as the estimated mean of *Y_FW_* on MD+1 is 40% lower compared with athletes’ best scores and is significantly different from the same index detected on MD (Figure 2b). Therefore, similar to MS, the *Y_FW_* value varied 24 h after the match and tended to return to its maximum in the days closest to the next match; this finding is consistent with other studies conducted on young rugby players [1,20,21,25,28]. Regarding the Mo dimension, the data relating to *Y_Mo_* show a stable average across all the time points analyzed, as shown in Figure 2b, where a consistently flat trend is displayed; the estimated mean *Y_Mo_* value associated with MD+2 is 20% lower than the athletes’ best scores but is also the best value during FSN18, demonstrating that MD-mediated stress decreases in most athletes (Supplementary Materials, Table S1). Indeed, the international match day is a potentially stressful event [24], but this condition does not appear to negatively affect the athletes’ responses during the competition period. Additionally, *Y_Mo_* on MD+1 shows a significant and positive correlation with TD detected on MD, as reported in the results (Figure 4f), suggesting a positive association between match activity and post-match mood. As for SQ, at 24 h post-match, the estimated mean *Y_SQ_* value is 25% lower than the athletes’ best scores, whereas at all other time points analyzed, the mean *Y_SQ_* value increased (Supplementary Materials, Table S1). Although small variations were detected, no statistically significant differences were observed. Findings from the study by Carlos A. Ramirez et al., conducted during international youth tournaments, showed that both sleep quality and quantity contribute to improved athletic performance [29]. Therefore, this dimension could be a useful parameter for evaluating athletes’ readiness [23,25,28,29]. Nevertheless, including additional questions, such as bedtime, wake-up time, and wake after sleep onset, as described in the Core Consensus Sleep Diary and already applied by Ramirez et al., would be useful for an accurate assessment of sleep quantity [23]. However, including too many items in daily assessments may become overly demanding for athletes in terms of time and attention; hence, in the present study, a simplified and short tool was used to focus mainly on its purpose. Despite this simplified approach, our findings are consistent with Ramirez’s study, supporting the potential importance of sleep monitoring and optimization during youth rugby competitions, as well as the role of sleep in athlete monitoring and performance.

### 4.2. Objective Components

As reported in the results section, although AST seems to vary with the athletes’ fatigue state, the two CTs considered in this study showed few significant differences between the time points analyzed (Table 3). However, their estimated mean values on MD+1 were the lowest in their respective curves, indicating a reduced readiness state 24 h after competition. Subsequently, as observed for the subjective components, both AST and SRT mean values increased, reaching their maximum, except in athletes presenting physical signs of reduced function, similarly to the measurements recorded during the training camp. In a previous review, Taylor et al. found that 61% of strength and conditioning coaches usually proposed strength-based, sport-specific practical tests to their athletes in order to assess readiness [22]. This may be explained by the fact that adductor strength is directly involved in rugby-specific actions such as sprinting, changes in direction, tackling, and physical contact [33,34,42], whereas the SRT primarily reflects flexibility characteristics [36,37] that may be less sensitive to short-term fatigue fluctuations. In addition, *Y_AST_* showed moderate and significant positive correlations with the 4-dRI and overall normalized indices (Figure 4a–d). These findings support that AST is an effective and simple to use tool for coaches in evaluating athletes’ readiness daily.

The development program for young international players includes daily strength and speed training; therefore, monitoring readiness in terms of flexibility and strength may provide useful information regarding athletes’ physical condition [33,36,38,48]. However, Taylor et al. reported that 54% of athletic trainers use jump assessments to monitor internal load [22]; similarly, Ramirez’s study, conducted during FSN18 in 2018, assessed internal load through daily evaluation of morning jumps, where analysis showed a continuous increase in jump performance in subsequent matches, even exceeding reference values, due to a hypothetical influence of the “learning effect” [25,47], the process by which repetition of a new motor skill over short periods leads to rapid improvements in performance, which become smaller as one becomes familiar with the action [47]. Conversely, the CTs applied in this study, although not sport-specific, are likely less susceptible to learning effects than repeated performance-based assessments, and their clinical relevance in assessing muscle function supports their inclusion within a multidimensional athlete-monitoring approach.

### 4.3. How Fatigue Influences Match Demands

In rugby, a competitive match usually represents the highest source of external load, often exceeding that accumulated during training sessions [17]. The relationship between MD GPS tracking data and *Y_MS_* obtained on MD+1, as displayed in the flexplots in Figure 3A–C, shows that players who covered over 4000 m, completed more than 20 sprint efforts, and played more than 40 min appeared to perceive a more fatigued state 24 h after the match. These exploratory observations suggest that higher match loads may be associated with a greater fatigue response 24 h after competition. Notably, similar to the MS dimension, particularly with regard to MP, the relationship between GPS tracking data on MD and 4-dRI and *Y_FW_* on MD+1 shows that players who played over 40 min were affected by fatigue upon waking 24 h after the match (Figure 3A and Figure 3F, respectively); such a finding supports that the relationship between match load and readiness on MD+1 may differ across dimensions and may become more evident at higher levels of match exposure, suggesting that higher match load is associated with lower readiness scores on the following day. Conversely, the Mo dimension shows an inverse trend line compared to MS and FW: *Y_Mo_* values on MD+1 tended to be higher in athletes exposed to greater match loads (Figure 3A–C) [27]. A different behavior was observed for the SQ dimension. Its trend line changes in relation to GPS metrics: athletes with a greater number of sprint efforts tended to show lower *Y_SQ_* values (Figure 3B), which could be due to a greater demand for strength during the specific neuromuscular effort of the rugby match [29]; on the other hand, the relationship between *Y_SQ_* and TD seems to favor sleep quality (*Y_SQ_* close to 0.8; Figure 3A), probably due to the fatigue accumulated after covering greater distances. As stated in previous studies, fatigue can alter physiological responses by affecting athletes’ sleep and behaviors [27,29], and this could explain the different relationship of *Y_SQ_* with nEF and TD. Finally, these descriptive trends suggest that higher GPS-derived external load metrics may be associated with lower readiness indices; however, these observations should be considered exploratory and require confirmation in future studies specifically designed to identify potential load-related thresholds.

Taken together, the present findings suggest that the 24 h period following competition may represent a critical window for monitoring readiness in youth rugby players. Lower 4-dRI and adductor squeeze test values were generally observed on MD+1, while athletes exposed to greater match demands tended to report lower readiness on the following day. In this context, combining subjective measures, clinically applicable tests, and GPS-derived match-load metrics may provide a practical approach for monitoring post-match recovery, although further research is required to define the most effective monitoring strategies and their practical application in team sports.

## 5. Limitations of the Study

A limitation of the present study concerns the use of equal weighting in the construction of the composite 4-dRI. Although all indices were normalized on a 0–1 scale [49] and contributed meaningfully to monitoring athletes’ readiness, the observed differences in sensitivity among dimensions, particularly for fatigue upon waking and muscle soreness, suggest that equal weighting may not optimally reflect their relative contribution to readiness. Future research should investigate alternative weighting approaches to enhance the accuracy and interpretability of composite indices.

Furthermore, the present findings were obtained from a single cohort of youth rugby players competing in an international tournament setting. Future studies involving larger samples from different teams and competitive contexts are needed to further evaluate the robustness of the proposed monitoring approach. In addition, only male athletes were included in the present investigation; however, future research should examine the applicability of this monitoring approach in female rugby players, as readiness responses may be influenced by menstrual-cycle-related physiological fluctuations and other sex-specific factors [50,51,52].

## 6. Conclusions

From a practical perspective, the day following competition appears to be a key time point for identifying players experiencing the greatest fatigue response. Athletes exposed to higher match demands generally reported lower readiness on MD+1, suggesting that recovery monitoring should be prioritized during the first 24 h after match play. Among the investigated dimensions, fatigue upon waking and muscle soreness appeared particularly sensitive to post-match changes in readiness. Nevertheless, further considerations regarding the aggregation process, weighting strategies, and the robustness of such composite indices are needed. Future studies should also assess the applicability of this approach across different youth-team settings, evaluate the integration of additional psychological and psychophysiological assessments, and investigate its relationship with performance outcomes and injury risk.

## Figures and Tables

**Table 1:** Pairwise comparisons of subjective indices across match days (linear mixed-effects models).

Variable	Comparison	Estimate	SE	df	t	*p* †
4-dRI	MD vs. MD+1	0.093	0.019	24.374	4.793	<0.001 ***
	MD vs. MD+2	0.004	0.012	36.998	0.303	1.000
	MD vs. MD+3	0.038	0.014	31.534	2.827	0.049 *
	MD+1 vs. MD+2	−0.090	0.016	30.037	−5.611	<0.001 ***
	MD+1 vs. MD+3	−0.055	0.014	34.116	−3.848	0.003 **
	MD+2 vs. MD+3	0.035	0.012	27.517	2.838	0.051
*Y_FW_*	MD vs. MD+1	0.176	0.032	23.485	5.428	<0.001 ***
	MD vs. MD+2	0.049	0.023	34.474	2.113	0.252
	MD vs. MD+3	0.100	0.026	34.525	3.873	0.003 **
	MD+1 vs. MD+2	−0.127	0.027	30.191	−4.748	<0.001 ***
	MD+1 vs. MD+3	−0.077	0.026	40.350	−2.929	0.033 *
	MD+2 vs. MD+3	0.050	0.024	72.163	2.102	0.234
*Y_Mo_*	MD vs. MD+1	−0.003	0.019	130.541	−0.148	1.000
	MD vs. MD+2	−0.039	0.019	83.550	−1.983	0.304
	MD vs. MD+3	−7.625 × 10^−4^	0.023	28.395	−0.033	1.000
	MD+1 vs. MD+2	−0.036	0.021	125.877	−1.714	0.534
	MD+1 vs. MD+3	0.002	0.024	32.871	0.085	1.000
	MD+2 vs. MD+3	0.038	0.023	40.669	1.659	0.629
*Y_SQ_*	MD vs. MD+1	0.070	0.029	24.661	2.405	0.144
	MD vs. MD+2	−0.012	0.020	24.386	−0.606	1.000
	MD vs. MD+3	0.008	0.019	36.010	0.403	1.000
	MD+1 vs. MD+2	−0.082	0.031	25.373	−2.677	0.077
	MD+1 vs. MD+3	−0.062	0.024	25.523	−2.654	0.081
	MD+2 vs. MD+3	0.020	0.023	28.313	0.848	1.000
*Y_MS_*	MD vs. MD+1	0.126	0.027	22.926	4.651	<0.001 ***
	MD vs. MD+2	0.021	0.021	39.664	0.967	1.000
	MD vs. MD+3	0.047	0.021	41.417	2.201	0.200
	MD+1 vs. MD+2	−0.106	0.024	35.322	−4.432	<0.001 ***
	MD+1 vs. MD+3	−0.079	0.023	49.510	−3.414	0.008 **
	MD+2 vs. MD+3	0.027	0.021	117.767	1.250	1.000

Note: Estimates represent differences in estimated marginal means. * *p* < 0.05, ** *p* < 0.01, *** *p* < 0.001. † Bonferroni-adjusted p-values for multiple pairwise comparisons. Abbreviations: MD, match day; *Y_FW_*, normalized fatigue upon waking; *Y_Mo_*, normalized mood; *Y_SQ_*, normalized sleep quality; *Y_MS_*, normalized muscle soreness; 4-dRI, four-dimension readiness index.

**Table 2:** Spearman’s rank correlation coefficients (ρ) among subjective indices and the composite 4-dRI.

Subjective Indices			Spearman’s Rho	*p*
*Y_FW_*	-	*Y_Mo_*	0.295 ***	<0.001
*Y_FW_*	-	*Y_SQ_*	0.180 **	0.007
*Y_FW_*	-	*Y_MS_*	0.456 ***	<0.001
*Y_FW_*	-	4-dRI	0.739 ***	<0.001
*Y_Mo_*	-	Ysq	0.278 ***	<0.001
*Y_Mo_*	-	*Y_MS_*	0.207 **	0.002
*Y_Mo_*	-	4-dRI	0.617 ***	<0.001
*Y_SQ_*	-	*Y_MS_*	0.146 *	0.029
*Y_SQ_*	-	4-dRI	0.498 ***	<0.001
*Y_MS_*	-	4-dRI	0.728 ***	<0.001

Abbreviations: *Y_FW_*, normalized fatigue upon waking; *Y_Mo_*, normalized mood; *Y_SQ_*, normalized sleep quality; *Y_MS_*, normalized muscle soreness; 4-dRI, four-dimension readiness index. * *p* < 0.05, ** *p* < 0.01, *** *p* < 0.001.

**Table 3:** Pairwise comparisons of objective indices across match days (linear mixed-effects models).

Variable	Comparison	Estimate	SE	df	t	*p* †
YAST	MD vs. MD+1	0.032	0.010	33.928	3.319	0.013 *
	MD vs. MD+2	0.008	0.009	92.302	0.902	1.000
	MD vs. MD+3	0.006	0.011	25.959	0.536	1.000
	MD+1 vs. MD+2	−0.024	0.010	25.243	−2.300	0.180
	MD+1 vs. MD+3	−0.026	0.010	29.168	−2.612	0.084
	MD+2 vs. MD+3	−0.002	0.012	27.121	−0.144	1.000
YSRT	MD vs. MD+1	0.017	0.008	30.184	2.183	0.222
	MD vs. MD+2	−0.010	0.007	61.724	−1.577	0.719
	MD vs. MD+3	−0.004	0.007	28.812	−0.538	1.000
	MD+1 vs. MD+2	−0.028	0.009	24.625	−2.950	0.041 *
	MD+1 vs. MD+3	−0.021	0.009	25.779	−2.390	0.147
	MD+2 vs. MD+3	0.007	0.008	28.096	0.828	1.000

Note: Estimates represent differences in estimated marginal means. * *p* < 0.05. † Bonferroni-adjusted p-values for multiple pairwise comparisons. Abbreviations: MD, match day; *Y_AST_*, normalized adductor squeeze test; *Y_SRT_*, normalized sit-and-reach test.

## Data Availability

The data presented in this study are not publicly available due to privacy and ethical restrictions but may be available upon reasonable request and with permission from the Italian Rugby Federation (FIR).

## Supplementary Materials

The following supporting information can be downloaded at: https://www.mdpi.com/article/10.3390/sports14070288/s1, Table S1: Descriptive Statistics of the subjective part and objective part (Match Day [MD], 24 h post-match [MD+1], 48 h post-match [MD+2], and 72 h post-match [MD+3]); Table S2: Descriptive statistics of GPS-derived variables collected on Match Days (MD1, MD2, and MD3); Figure S1: Readiness questionnaire used for the subjective component. The value “1” represents the worst perceived subjective condition, while the value “5” represents the best; Figure S2: Field tests (FT): the Adductor Squeeze Test (AST) and the Sit-and-Reach Test (SRT). Both tests were conducted every morning before breakfast in a dedicated room using a self-assessment procedure.

## Abbreviations

The following abbreviations are used in this manuscript:

FSN18	Six Nations Under-18 Festival
U18	Under-18 rugby teams
MD	Match day
MD+1	One day post-match
MD+2	Two days post-match
MD+3	Three days post-match
FW	Fatigue upon waking
Mo	Mood
SQ	Sleep quality
MS	Muscle soreness
AST	Adductor Squeeze Test
SRT	Sit-and-Reach Test
YFW	Normalized fatigue upon waking
*Y_Mo_*	Normalized mood
*Y_SQ_*	Normalized sleep quality
*Y_MS_*	Normalized muscle soreness
*Y_AST_*	Normalized adductor squeeze test
*Y_SRT_*	Normalized sit-and-reach test
4-dRI	Four-dimensional readiness index
GPS	Global Positioning System
TD	total distance
nEF	Number of high-speed efforts (>20 km·h^−1^)
MP	minutes played
CTs	Clinical tests
LMM	Linear mixed-effects model
LOWESS	Locally weighted scatterplot smoothing

## References

[1] Lacome M., Carling C., Hager J.-P., Dine G., Piscione J. (2018). Workload, Fatigue, and Muscle Damage in an Under-20 Rugby Union Team Over an Intensified International Tournament. Int. J. Sports Physiol. Perform..

[2] Yeomans C., Kenny I.C., Cahalan R., Warrington G.D., Harrison A.J., Hayes K., Lyons M., Campbell M.J., Comyns T.M. (2018). The Incidence of Injury in Amateur Male Rugby Union: A Systematic Review and Meta-Analysis. Sports Med..

[3] Roe G. (2017). Changes in markers of fatigue following a competitive match in elite academy rugby union players. SA J. Sports Med..

[4] Twist C., Highton J. (2013). Monitoring Fatigue and Recovery in Rugby League Players. Int. J. Sports Physiol. Perform..

[5] Hulin B.T., Gabbett T.J., Lawson D.W., Caputi P., Sampson J.A. (2016). The acute:chronic workload ratio predicts injury: High chronic workload may decrease injury risk in elite rugby league players. Br. J. Sports Med..

[6] Williams S., Trewartha G., Kemp S., Stokes K. (2013). A Meta-Analysis of Injuries in Senior Men’s Professional Rugby Union. Sports Med..

[7] Laaksonen J., Vaajala M., Pakarinen O., Liukkonen R., Kuitunen I. (2026). Epidemiology of rugby injuries: A systematic review and meta-analysis. BMJ Open Sport Exerc. Med..

[8] Emery C.A., Roy T.-O., Whittaker J.L., Nettel-Aguirre A., Van Mechelen W. (2015). Neuromuscular training injury pre-vention strategies in youth sport: A systematic review and meta-analysis. Br. J. Sports Med..

[9] Gabbett T.J. (2016). The training—Injury prevention paradox: Should athletes be training smarter and harder?. Br. J. Sports Med..

[10] Tee J.C., Lambert M.I., Coopoo Y. (2016). GPS comparison of training activities and game demands of professional rugby union. Int. J. Sports Sci. Coach..

[11] Marinescu G., Dreve A. (2019). Identification of Effort Parameters in a Rugby Match with the Gps.

[12] Reardon C., Tobin D.P., Delahunt E. (2015). Application of Individualized Speed Thresholds to Interpret Position Specific Running Demands in Elite Professional Rugby Union: A GPS Study. PLoS ONE.

[13] Beenham M., Barron D.J., Fry J., Hurst H.H., Figueirdo A., Atkins S. (2017). A Comparison of GPS Workload Demands in Match Play and Small-Sided Games by the Positional Role in Youth Soccer. J. Hum. Kinet..

[14] Pillitteri G., Giustino V., Petrucci M., Rossi A., Bellafiore M., Thomas E., Iovane A., Bianco A., Palma A., Battaglia G. (2023). External load profile during different sport-specific activities in semi-professional soccer players. BMC Sports Sci. Med. Rehabil..

[15] Read D.B., Jones B., Phibbs P.J., Roe G.A.B., Darrall-Jones J., Weakley J.J.S., Till K. (2018). The physical characteristics of match-play in English schoolboy and academy rugby union. J. Sports Sci..

[16] Gabbett T.J. (2013). Influence of playing standard on the physical demands of professional rugby league. J. Sports Sci..

[17] Moody J. (2022). Positional Demands of a Tier 2 International Rugby Union Team using GPS Metrics, Match Performance Indicators and Worst-Case Scenarios. Res. Investig. Sports Med..

[18] Al Haddad H., Méndez-Villanueva A., Torreño N., Munguía-Izquierdo D., Suárez-Arrones L. (2018). Variability of GPS-derived running performance during official matches in elite professional soccer players. J. Sports Med. Phys. Fit..

[19] Baptista I., Alexandersen A., Winther A.K., Johansen D., Pettersen S.A. (2025). Effect of match load on perceived wellness in highly trained female football players. PLoS ONE.

[20] Saw A.E., Main L.C., Gastin P.B. (2016). Monitoring the athlete training response: Subjective self-reported measures trump commonly used objective measures: A systematic review. Br. J. Sports Med..

[21] Starling L.T., Lambert M.I. (2018). Monitoring Rugby Players for Fitness and Fatigue: What Do Coaches Want?. Int. J. Sports Physiol. Perform..

[22] Taylor K.L., Chapman D.W., Cronin J.B., Newton M.J., Gill N.D. (2012). Fatigue monitoring in high performance sport: A survey of current trends. J. Aust. Strength Cond..

[23] Carney C.E., Buysse D.J., Ancoli-Israel S., Edinger J.D., Krystal A.D., Lichstein K.L., Morin C.M. (2012). The Consensus Sleep Diary: Standardizing Prospective Sleep Self-Monitoring. Sleep.

[24] Drole K., Doupona M., Steffen K., Jerin A., Paravlic A. (2025). Associations between subjective and objective measures of stress and load: An insight from 45-week prospective study in 189 elite athletes. Front. Psychol..

[25] Ramírez-López C., Till K., Sawczuk T., Giuliano P., Peeters A., Beasley G., Murray F., Pledger S., Read D., Jones B. (2020). A multi-nation examination of the fatigue and recovery time course during the inaugural Under-18 Six Nations rugby union competition. J. Sports Sci..

[26] Jones C.M., Griffiths P.C., Mellalieu S.D. (2017). Training Load and Fatigue Marker Associations with Injury and Illness: A Systematic Review of Longitudinal Studies. Sports Med..

[27] Tornero-Aguilera J.F., Jimenez-Morcillo J., Rubio-Zarapuz A., Clemente-Suárez V.J. (2022). Central and Peripheral Fatigue in Physical Exercise Explained: A Narrative Review. Int. J. Environ. Res. Public Health.

[28] Ramírez-López C., Till K., Weaving D., Boyd A., Peeters A., Beasley G., Bradley S., Giuliano P., Venables C., Jones B. (2022). Does perceived wellness influence technical–tactical match performance? A study in youth international rugby using partial least squares correlation analysis. Eur. J. Sport Sci..

[29] Ramírez C.A., Till K., Beasley G., Giuliano P., Leduc C., Dalton-Barron N., Weakley J.J., Jones B. (2020). Sleep patterns of elite youth team-sport athletes prior to and during international competition. Sci. Med. Footb..

[30] Dai X., Yan J., Bi X. (2025). Weekly Fluctuations in Subjective and Objective Measures of Internal Training Load and Their Relationships in Male Elite Rowers. J. Sports Sci. Med..

[31] Macedo A.G., Almeida T.A.F., Massini D.A., de Oliveira D.M., Espada M.C., Robalo R.A.M., Hernández-Beltrán V., Gamonales J.M., Terra A.M.S.V., Filho D.M.P. (2024). Load Monitoring Methods for Controlling Training Effectiveness on Physical Conditioning and Planning Involvement: A Narrative Review. Appl. Sci..

[32] Epp-Stobbe A., Tsai M.-C., Klimstra M.D. (2024). Predicting Athlete Workload in Women’s Rugby Sevens Using GNSS Sensor Data, Contact Count and Mass. Sensors.

[33] Hodgson L., Hignett T., Edwards K. (2015). Normative adductor squeeze tests scores in rugby. Phys. Ther. Sport.

[34] Moreno-Pérez V., Travassos B., Calado A., Gonzalo-Skok O., Del Coso J., Mendez-Villanueva A. (2019). Adductor squeeze test and groin injuries in elite football players: A prospective study. Phys. Ther. Sport.

[35] Ayala F., Sainz De Baranda P., De Ste Croix M., Santonja F. (2012). Fiabilidad y validez de las pruebas sit-and-reach: Revisión sistemática. Rev. Andal. Med. Deporte.

[36] Ayala F., Sainz De Baranda P., De Ste Croix M., Santonja F. (2012). Absolute reliability of five clinical tests for assessing hamstring flexibility in professional futsal players. J. Sci. Med. Sport.

[37] Mayorga-Vega D., Merino-Marban R., Viciana J. (2014). Criterion-Related Validity of Sit-And-Reach Tests for Estimating Hamstring and Lumbar Extensibility: A Meta-Analysis. J. Sports Sci. Med..

[38] Edouard P., Pollock N., Guex K., Kelly S., Prince C., Navarro L., Branco P., Depiesse F., Gremeaux V., Hollander K. (2022). Hamstring Muscle Injuries and Hamstring Specific Training in Elite Athletics (Track and Field) Athletes. Int. J. Environ. Res. Public Health.

[39] Mazziotta M., Pareto A. (2022). Weighting in composite indices construction: The case of the Mazziotta-Pareto index. Riv. Ital. Econ. Demogr. Stat..

[40] Guitart M., Casals M., Casamichana D., Cortés J., Valle F.X., McCall A., Cos F., Rodas G. (2022). Use of GPS to measure external load and estimate the incidence of muscle injuries in men’s football: A novel descriptive study. PLoS ONE.

[41] Jebb A.T., Ng V., Tay L. (2021). A Review of Key Likert Scale Development Advances: 1995–2019. Front. Psychol..

[42] DeLang M.D., Garrison J.C., Hannon J.P., Ishøi L., Thorborg K. (2023). Weekly screening of youth male football players: A 14-week longitudinal investigation of interactions between groin pain and long lever adductor squeeze strength. J. Sci. Med. Sport.

[43] Read D.B., Jones B., Phibbs P.J., Roe G.A., Darrall-Jones J.D., Weakley J.J., Till K. (2017). Physical Demands of Representative Match-Play in Adolescent Rugby Union. J. Strength Cond. Res..

[44] Oleksy Ł., Mika A., Królikowska A., Kuchciak M., Stolarczyk M., Kielnar R., Racheniuk H., Szczegielniak J., Łuszczki E., Stolarczyk A. (2021). Composite Score of Readiness (CSR) as Holistic Profiling of Functional Deficits in Footballers Following ACL Reconstruction. J. Clin. Med..

[45] Urrutia S., Cappuccio Á., González-Ramírez A. (2024). Ecuación para el control de la carga de entrenamiento con datos de GPS en fútbol de alto rendimiento (Equation for player load control of training with GPS in a high-performance soccer). Retos.

[46] Zanin M., Azzalini A., Ranaweera J., Weaving D., Darrall-Jones J., Roe G. (2023). The contributing external load factors to internal load during small-sided games in professional rugby union players. Front. Sports Act. Living.

[47] Lund H., Søndergaard K., Zachariassen T., Christensen R., Bülow P., Henriksen M., Bartels E.M., Danneskiold-Samsøe B., Bliddal H. (2005). Learning effect of isokinetic measurements in healthy subjects, and reliability and comparability of Biodex and Lido dynamometers. Clin. Physiol. Funct. Imaging.

[48] Castro-Piñero J., Chillón P., Ortega F.B., Montesinos J.L., Sjöström M., Ruiz J.R. (2009). Criterion-Related Validity of Sit-and-Reach and Modified Sit-and-Reach Test for Estimating Hamstring Flexibility in Children and Adolescents Aged 6–17 Years. Int. J. Sports Med..

[49] Greco S., Ishizaka A., Tasiou M., Torrisi G. (2019). On the Methodological Framework of Composite Indices: A Review of the Issues of Weighting, Aggregation, and Robustness. Soc. Indic. Res..

[50] Giuliano P., Scalia D., Cè E., Barachetti L. (2026). Integrated readiness monitoring based on post-competition fatigue in team sports: A longitudinal study comparing male and female athletes. Int. Sports Stud..

[51] Roffler A., Fleddermann M.-T., De Haan H., Krüger K., Zentgraf K. (2024). Menstrual cycle tracking in professional volleyball athletes. Front. Sports Act. Living.

[52] Carmichael M.A., Thomson R.L., Moran L.J., Wycherley T.P. (2021). The Impact of Menstrual Cycle Phase on Athletes’ Performance: A Narrative Review. Int. J. Environ. Res. Public Health.

