# Supplementary material for: Monitoring Match-Related Fatigue in Youth Rugby Players Using a Readiness Index and Clinical Tests
Source: Sports (Basel). 2026 Jul 8;14(7):288. doi: 10.3390/sports14070288 (PMC13418244; doi:10.3390/sports14070288)

Supplementary Materials:

Table S1. Descriptive Statistics of the subjective part and objective part (Match Day [MD], 24 h post-match [MD+1], 48 h post-match [MD+2], and 72 h post-match [MD+3]).

Variables	days	Median	IQR	Mean	Std. Deviation
Y_{FW}	MD	0.750	0.250	0.806	0.164
Y_{FW}	MD+1	0.600	0.250	0.627	0.217
Y_{FW}	MD+2	0.750	0.118	0.759	0.155
Y_{FW}	MD+3	0.750	0.263	0.710	0.188
Y_{Mo}	MD	0.750	0.400	0.764	0.184
Y_{Mo}	MD+1	0.750	0.400	0.784	0.178
Y_{Mo}	MD+2	0.750	0.250	0.802	0.147
Y_{Mo}	MD+3	0.750	0.383	0.766	0.178
Y_{SQ}	MD	0.800	0.250	0.815	0.160
Y_{SQ}	MD+1	0.800	0.250	0.764	0.184
Y_{SQ}	MD+2	0.800	0.200	0.830	0.152
Y_{SQ}	MD+3	0.800	0.250	0.814	0.160
Y_{MS}	MD	0.600	0.350	0.640	0.193
Y_{MS}	MD+1	0.400	0.290	0.520	0.220
Y_{MS}	MD+2	0.600	0.388	0.630	0.215
Y_{MS}	MD+3	0.600	0.350	0.595	0.191
4-dRI	MD	0.750	0.140	0.757	0.120
4-dRI	MD+1	0.650	0.162	0.674	0.136
4-dRI	MD+2	0.730	0.182	0.756	0.121
4-dRI	MD+3	0.730	0.152	0.723	0.120
Yast	MD	0.830	0.145	0.807	0.117
Y_{AST}	MD+1	0.785	0.170	0.779	0.115
Y_{AST}	MD+2	0.810	0.165	0.803	0.122
Y_{AST}	MD+3	0.820	0.178	0.799	0.116
Y_{SRT}	MD	0.920	0.090	0.913	0.060
Y_{SRT}	MD+1	0.900	0.085	0.893	0.073
Y_{SRT}	MD+2	0.930	0.087	0.924	0.061
Y_{SRT}	MD+3	0.920	0.070	0.917	0.056
FW	MD	3.000	1.000	3.267	0.704
FW	MD+1	2.500	1.000	2.604	0.984
FW	MD+2	3.000	0.000	3.060	0.682
FW	MD+3	3.000	1.000	2.880	0.773
Mo	MD	3.000	0.000	3.160	0.806
Mo	MD+1	3.000	1.000	3.271	0.869
Mo	MD+2	3.000	0.750	3.300	0.614
Mo	MD+3	3.000	0.000	3.200	0.782
SQ	MD	4.000	1.000	3.747	0.737

Variables	days	Median	IQR	Mean	Std. Deviation
SQ	MD+1	4.000	1.000	3.521	0.875
SQ	MD+2	4.000	0.000	3.800	0.639
SQ	MD+3	4.000	1.000	3.740	0.751
MS	MD	3.000	1.000	2.947	0.868
MS	MD+1	2.000	1.000	2.375	0.981
MS	MD+2	3.000	1.000	2.840	0.912
MS	MD+3	3.000	1.000	2.740	0.876

Table S2. Descriptive statistics of GPS-derived variables collected on Match Days (MD1, MD2, and MD3).

GPS data	Match days	Median	IQR	Mean	Std. Deviation
MP	MD1	55.000	56.000	49.524	26.615
MP	MD2	42.000	31.000	46.640	21.647
MP	MD3	35.000	28.000	40.524	18.702
TD	MD1	4610.000	3783.000	4317.522	1958.898
TD	MD2	3123.000	2082.000	3730.840	1647.000
TD	MD3	2791.000	1747.000	3360.810	1345.038
nEF	MD1	11.000	13.000	11.435	8.575
nEF	MD2	9.000	8.000	12.720	10.530
nEF	MD3	14.000	12.000	14.333	9.851

Figure S1. Readiness questionnaire used for the subjective component. The value “1” represents the worst perceived subjective condition, while the value “5” represents the best.

How do you feel when you first wake up?

1. very tired
2. tired
3. normal
4. rested and refreshed
5. very rested and refreshed

What is your mood when you wake up?

1. exhausted/very irritated
2. stressed
3. normal
4. relaxed
5. excellent mood

How well did you sleep?

1. insomnia
2. tired and unwell
3. difficulty falling asleep
4. good
5. excellent

How sore do your muscles feel after physical activity?

1. very sore
2. slightly sore
3. not sore at all
4. feeling good
5. very energetic / feeling excellent

Figure S2. Field tests (FT): the Adductor Squeeze Test (AST) and the Sit-and-Reach Test (SRT). Both tests were conducted every morning before breakfast in a dedicated room using a self-assessment procedure.

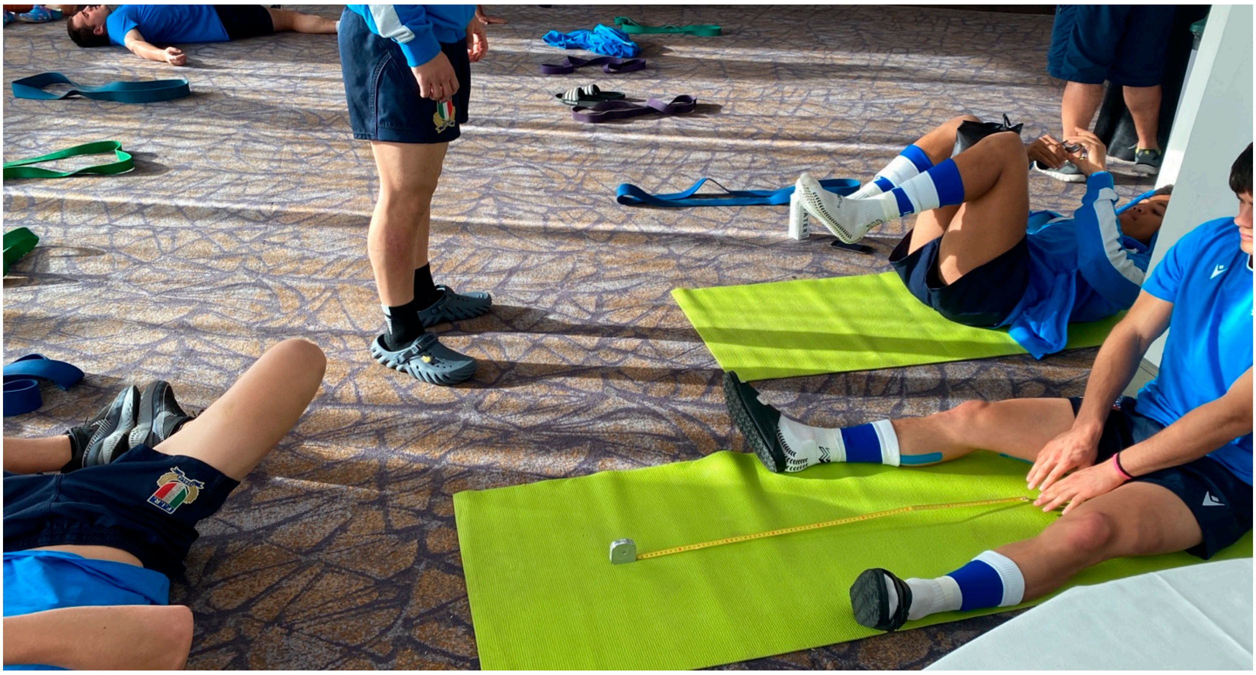

Supplement: Supplementary file 1 [file sports-14-00288-s001.zip › sports-4321556-supplementary.pdf]
